# Dynamics of Hyper-Reflective Foci and Hard Exudates in Diabetic Macular Edema Treated with Faricimab

**DOI:** 10.3390/jcm15166319

**Published:** 2026-08-15

**Authors:** Paul Widmann-Sedlnitzky, Kim Lien Huber, Irene Steiner, Markus Gumpinger, Heiko Stino, Tilman Schmoll, Ursula Schmidt-Erfurth, Stefan Sacu, Andreas Pollreisz

**Affiliations:** 1 Department of Ophthalmology, Medical University of Vienna, 1090 Vienna, Austria; 2 Institute of Medical Statistics, Center for Medical Data Science, Medical University of Vienna, 1090 Vienna, Austria; 3 Carl Zeiss Meditec AG, 73447 Oberkochen, Germany; 4 Department of Oncology, Center for Cancer Research, Medical University of Vienna, 1090 Vienna, Austria

**Keywords:** macular edema, diabetic retinopathy, tomography, optical coherence, biomarkers

## Abstract

**Background/Objectives:** Diabetic macular edema is caused by disruptions in the blood–retinal barrier, resulting in leakage and inflammation, appearing in optical coherence tomography as retinal fluid, hard exudates, and hyper-reflective foci. This study aimed to assess volumetric changes in hyper-reflective foci and hard exudates in faricimab-treated diabetic macular edema in real-world practice. **Methods:** This retrospective longitudinal study included forty-four eyes with faricimab-treated diabetic macular edema and follow-up of ≥40 weeks. Optical coherence tomography scans were analyzed at baseline, post-loading, 6 months and 1 year. Hyper-reflective foci and hard exudates were quantified across macular subfields using a machine-learning algorithm with manual correction. Outcomes included volumetric changes, best-corrected visual acuity, and central subfield thickness. Early morphologic responders were defined post-loading by ≥20% central subfield thickness reduction and/or central subfield thickness below 280 µm. **Results**: At 1 year, hyper-reflective foci and hard exudate volumes significantly decreased in the central subfield (median differences: −0.025 nL, *p* = 0.00028; −0.119 nL, *p* = 0.00025) and outer ring (−0.178 nL, *p* = 0.00041; −0.612 nL, *p* = 0.014). Hard exudate volume significantly decreased in the inner ring (−0.740 nL, *p* < 0.0001). Best-corrected visual acuity and central subfield thickness improved significantly (−0.10 logarithm of the minimum angle of resolution (logMAR), *p* = 0.0018; −48 µm, *p* < 0.0001). Total hyper-reflective foci volumes were similar between responders and non-responders. In contrast, hard exudate burden remained numerically higher in non-responders. **Conclusions:** During the year following the switch to faricimab, HRF and HE burden decreased alongside visual and anatomical improvement. HRF volumes were descriptively similar between early CST responders and non-responders, whereas HE burden remained numerically higher in non-responders.

## 1. Introduction

Diabetic macular edema (DME) is a leading cause of moderate to severe vision loss in developed countries [1]. Hyperglycemia triggers a cascade of angiogenic and inflammatory mediators, including vascular endothelial growth factor (VEGF) and angiopoietin-2 (Ang-2), increasing vascular permeability, disrupting the blood–retinal barrier, and causing intraretinal fluid accumulation [2].

Hyper-reflective retinal foci (HRF) are small, discrete lesions visible on optical coherence tomography (OCT) that vary in size and reflectivity. First described as extravasated lipoproteins and/or proteins, potentially serving as precursors to hard exudates, they have since been subclassified into vascular, exudative, pigmentary, and inflammatory HRF [3,4]. This classification differentiates structural features such as lipid or protein deposits, migrating retinal pigment epithelium cells, and activated microglia, enabling more precise interpretation of HRF as potential retinal biomarkers. Hard exudates (HEs) consist of lipid-rich and proteinaceous deposits, including fibrinogen and albumin, that leak from a compromised blood–retinal barrier. Similar to HRF, they are primarily localized in the outer plexiform layer. If left untreated, foveal HE can cause irreversible vision loss [5,6].

Currently, the gold standard for first-line DME treatment is repeated intravitreal injections of anti-VEGF agents [7,8]. These agents have shown efficacy in decreasing both the area of HE and the number of HRF in eyes with DME [9,10].

In September 2022, the bispecific antibody faricimab (Vabysmo, Genentech, San Francisco, CA, USA), inhibiting both VEGF-A and Ang-2, was approved in Europe for DME. Real-world studies have demonstrated strong morphological responses and potentially longer treatment intervals [11,12,13].

However, data on volumetric changes in HRF and HE under faricimab are limited. As HRF and HE may provide insights into the mechanisms of dual VEGF-A/Ang-2 inhibition and support individualized management, understanding their dynamics is essential.

This study aims to assess intraretinal HRF and HE dynamics in eyes with DME over one year following a switch to intravitreal faricimab in a real-world setting.

## 2. Methods

### 2.1. Patient Selection

In this retrospective, clinical longitudinal study, patients with DME switched from aflibercept to faricimab between July 2023 and October 2024 at the Department of Ophthalmology at the Medical University of Vienna were identified using the electronic medical record (VIBES registry). Ethical approval was granted by the ethics committee at the Medical University of Vienna (EK-Nr. 2095/2018), ensuring compliance with the Declaration of Helsinki. Written informed consent was obtained from all participating patients for the inclusion of their data and subsequent analyses.

Eyes treated with intravitreal faricimab for DME were screened using the following inclusion criteria:

- Presence of DME and HE in OCT at baseline.
- Follow-up of ≥40 weeks.
- No intravitreal steroid treatment within six months or anti-VEGF injection within four weeks before baseline.
- No switch to another agent within 40 weeks after baseline.
- Absence of other retinal diseases.
- No vitreoretinal surgery within 12 months before baseline.
- No cataract surgery within 6 months before baseline.
- Availability of Cirrus OCT (Carl Zeiss Meditec, Dublin, CA, USA) at baseline and at least one follow-up timepoint.

If both met the inclusion criteria, only the right eye was included. HRF/HE were assessed at baseline (visit 1), 4 weeks after the loading phase (visit 2), 6 months (visit 3), and 1 year (visit 4). Because patients were managed using individualized treat-and-extend (T&E) regimens, the study time points for visits 2–4 were assigned to the patient visit closest to the predefined interval.

### 2.2. Clinical Assessment and Imaging

The electronic medical record was used to extract imaging and clinical data at each time point, including best-corrected visual acuity (BCVA), 6 × 6 mm OCT imaging (Macular Cube 512 × 128, Cirrus 5000, Carl Zeiss Meditec Inc., Dublin, CA, USA) automated central subfield thickness (CST), color fundus photography (200° ultra-wide-field, Optomap, Nikon Co. Ltd., Tokyo, Japan) and the timing and number of faricimab injections. All patients received a loading dose of two monthly faricimab injections followed by a treat-and-extend (T&E) regimen with 2- to 4-week extensions at the discretion of the treating ophthalmologist.

### 2.3. Imaging Analysis

HRF and HE were segmented on OCT using a previously validated, machine-learning-based algorithm developed at the Medical University of Vienna [14]. The segmentations were reviewed and manually corrected by two expert graders (PWS and KLH) using internally developed, non-commercial annotation software. False-positive annotations were removed and missed lesions were added. OCT volumes with insufficient scan quality or truncated scans were excluded. The reflectivity of the retinal pigment epithelium served as the reference threshold for lesion identification. Hyper-reflective structures < 30 µm in diameter without back-shadowing were classified as HRF, whereas structures ≥ 30 µm with back-shadowing were classified as HE, consistent with previous studies [4,14,15]. HRF were excluded when located outside the neurosensory retina, within normally hyper-reflective layers (nerve fiber layer, external limiting membrane, ellipsoid zone, or interdigitation zone), within cystic septa, or in association with retinal vessels. The fovea was manually marked in each volume to ensure accurate Early Treatment Diabetic Retinopathy Study (ETDRS) grid placement. HRF and HE volume and count were determined for each ETDRS subfield.

### 2.4. Primary and Secondary Outcomes

The main outcome was the change in HRF/HE volume and number in each ETDRS subfield, and in the inner and outer rings, from baseline to visits 2, 3, and 4. Changes in HRF/HE metrics were correlated to CST, BCVA, and number of faricimab injections. Morphologic responders and non-responders at visit 2 (4 weeks after loading) were defined by CST: responders had a ≥20% CST reduction versus baseline and/or CST < 280 µm; all others were classified as non-responders.

### 2.5. Statistical Analysis

Metric variables are described as median (first quartile (Q1); third quartile (Q3)) and categorical variables as absolute frequencies and percentages. For visits 2–4, Wilcoxon signed-rank tests were used to compare HRF volume, HE volume, HRF number, HE number, BCVA, and CST versus baseline for each area (9 ETDRS subfields and inner/outer rings).

Spearman’s rank correlation coefficients (r_s_) were calculated for each area and time point to assess correlations between HRF/HE volume and BCVA, CST, and number of injections. For the following variables, the two morphologic response groups were descriptively compared: BCVA and the volume of HRF/HE in the central subfield, inner/outer rings, and in the total OCT volume.

Analyses were performed with R 4.4.0 [16]. Due to the exploratory study design, no adjustment for multiple testing was applied. The interpretation of the *p* values is exploratory.

## 3. Results

### 3.1. Patient Population and Baseline Characteristics

A retrospective assessment of consecutive DME patients switched from aflibercept to intravitreal faricimab between July and October 2024 was performed. Forty-four eyes of 44 patients met the inclusion criteria. Baseline characteristics are summarized in Table 1.

### 3.2. Visit Timepoints

The mean ± standard deviation (minimum, maximum) time from baseline to each study visit was 10.10 ± 1.82 (7, 15) weeks at visit 2, 24.74 ± 2.20 (20, 29) weeks at visit 3, and 49.47 ± 4.00 (41, 56) weeks at visit 4.

### 3.3. Morphology and Visual Acuity

At baseline, median (Q1; Q3) BCVA was 0.3 logarithm of the minimum angle of resolution (logMAR) (0.2 logMAR; 0.43 logMAR) and median CST was 351.5 µm (303 µm; 421.75 µm). BCVA significantly improved from baseline at all follow-up time points, with median reductions in logMAR scores of −0.1 (−0.15; 0) at visit 2 (*p* = 0.0018, *n* = 43), −0.1 (−0.2; 0) at visit 3 (*p* = 0.00055, *n* = 43) and −0.1 (−0.2; 0) at visit 4 (*p* = 0.0018, *n* = 44). Similarly, CST significantly decreased at all visits, with median reductions of −44 (−83; −27) µm at visit 2 (*p* < 0.0001, *n* = 41), −38 (−79; −4) µm at visit 3 (*p* = 0.001, *n* = 44) and −48 (−89; −5) µm at visit 4 (*p* < 0.0001, *n* = 43).

### 3.4. Intravitreal Injections and Treatment Interval

At visits 2, 3, and 4, patients received a median of 2 (2; 2), 3 (3; 4), and 6 (4.75; 6.25) faricimab injections, respectively. At visit 4, 64% of patients were on ≥10-week intervals, 50% on ≥16-week intervals, and 34% on 20-week intervals.

### 3.5. HRF and HE Dynamics

**Figure 1:**
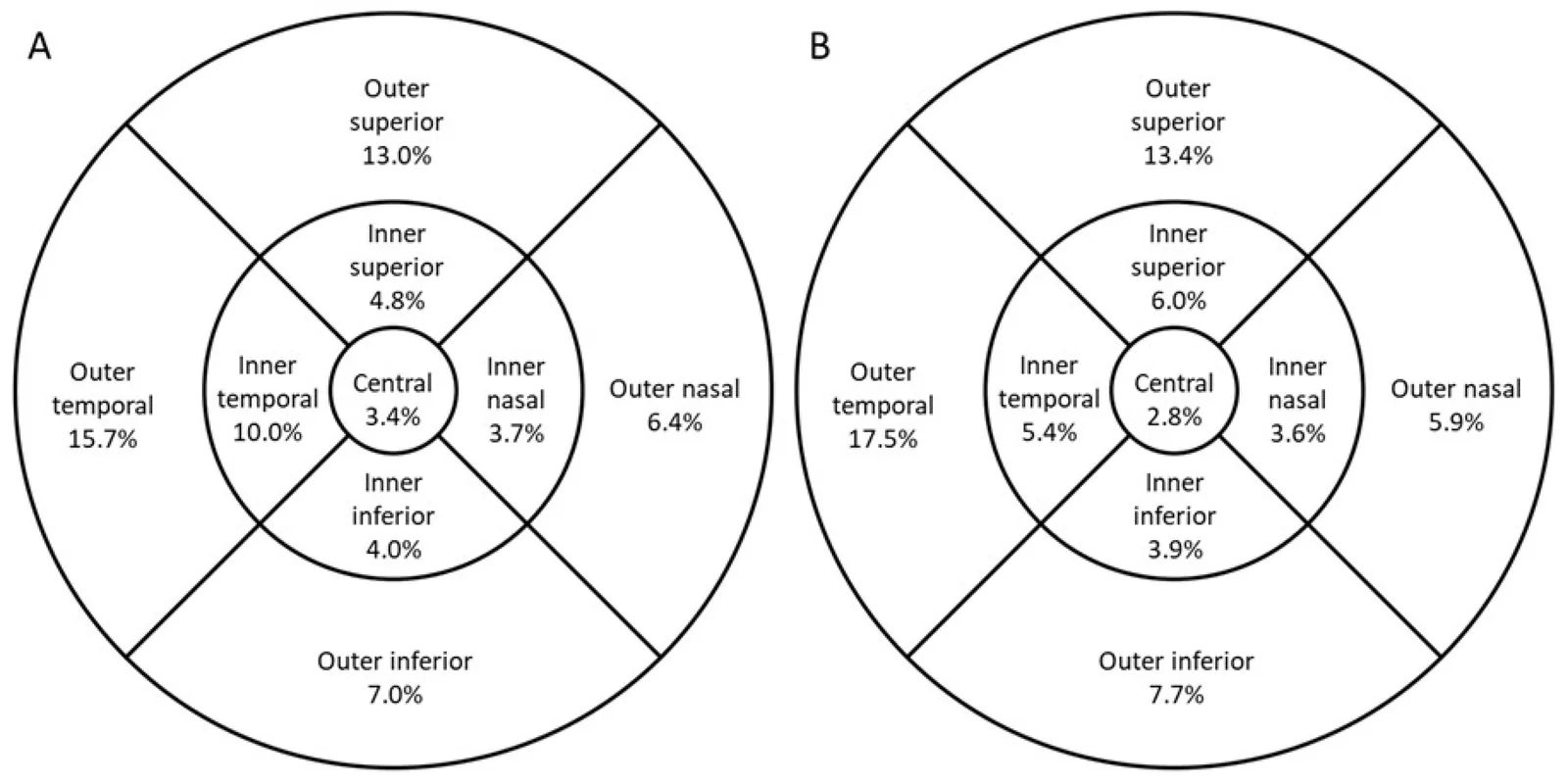
Median combined HRF/HE volume in each ETDRS subfield at baseline (**A**) and at 1 year (visit 4 (**B**)). For each eye, subfield volumes were expressed as percentages of the total macular HRF/HE volume, after which the median was calculated independently for each subfield. Consequently, the median percentages displayed are not expected to sum to 100%. Sample sizes were *n* = 41 at baseline and *n* = 40 at visit 4. Together, the four inner and four outer subfields constitute the inner and outer ETDRS rings, respectively.

Figure 1 shows the median combined HRF/HE volume distribution across the ETDRS subfields at baseline and visit 4. At baseline, the outer temporal (15.7%) and outer superior (13.0%) subfields showed the highest HRF/HE volumes. At visit 4, these remained the most affected regions (outer temporal 17.5%, outer superior 13.4%).

Baseline median (Q1; Q3) HRF volumes were 0.05 (0.03; 0.08) nL in the central subfield, 0.28 (0.19; 0.44) nL in the inner ring, and 0.61 (0.40; 0.92) nL in the outer ring. Corresponding median HE volumes were 0.21 (0.03; 0.39) nL, 1.54 (0.75; 3.64) nL, and 2.91 (0.66; 7.83) nL, respectively. Baseline median HRF and HE counts were 10.0 (5.0; 13.0) HRF and 4.0 (1.0; 9.0) HE in the central subfield, 54.0 (39.0; 77.0) HRF and 27.0 (10.0; 48.0) HE in the inner ring, and 126.0 (84.0; 166.0) HRF and 49.0 (14.0; 89.0) HE in the outer ring. Full baseline data for all subfields are provided in Supplementary Table S1.

**Figure 2:**
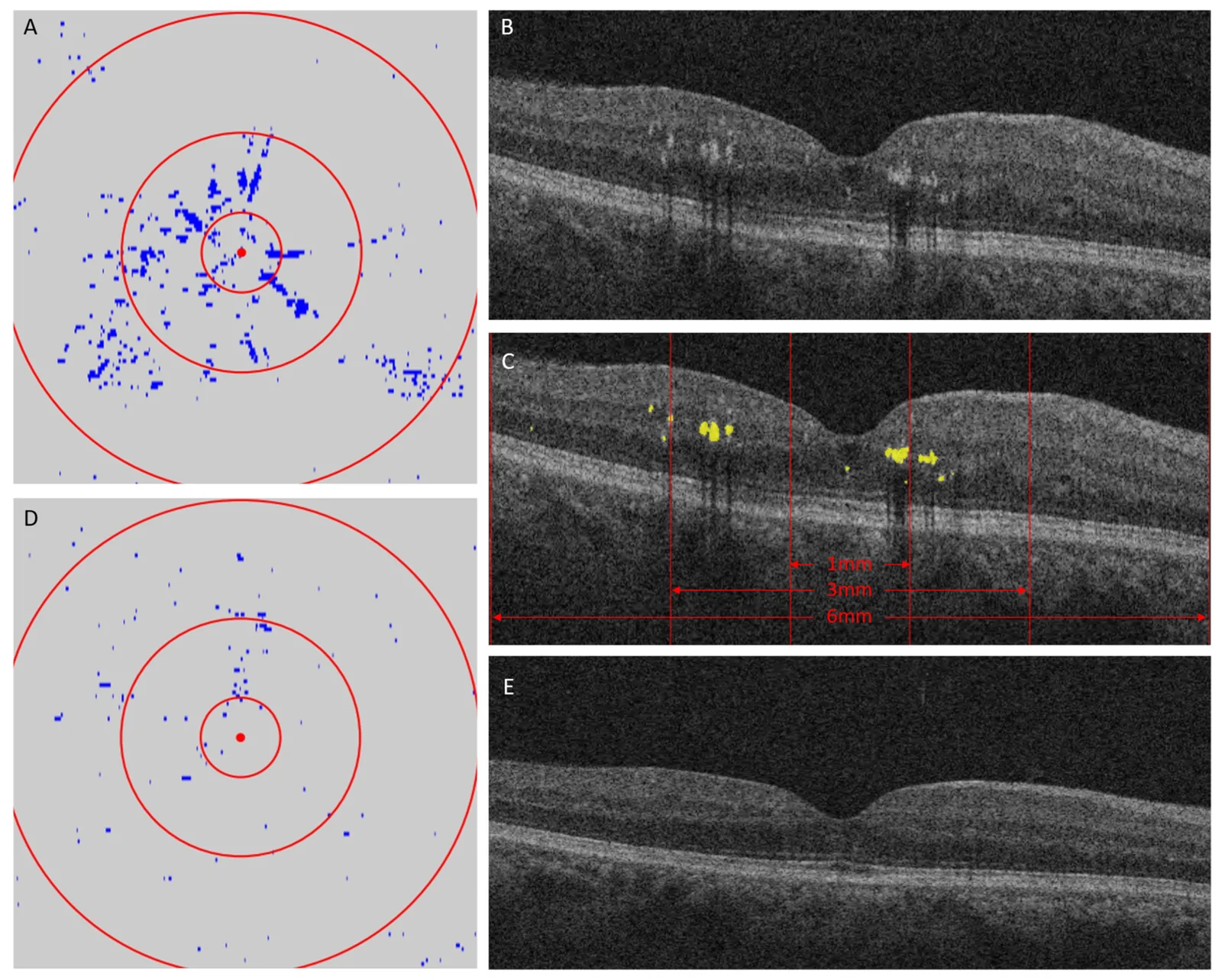
Case of a 45-year-old male patient with severe diabetic retinopathy: En-face projection and b-scans of HRF/HE at baseline (**A**–**C**) and at visit 4 (**D**,**E**). B-Scans are seen before (**B**) and after annotation (**C**), including the projection of the ETDRS rings.

At visit 4, HRF volume significantly decreased in the central, outer nasal, outer superior and outer temporal subfields as well as the outer ring. HE volume significantly decreased in the central, inner superior, inner and outer inferior, inner and outer temporal subfields and in both rings. A representative patient case demonstrating significant changes in HRF and HE volumes is presented in Figure 2.

**Figure 3:**
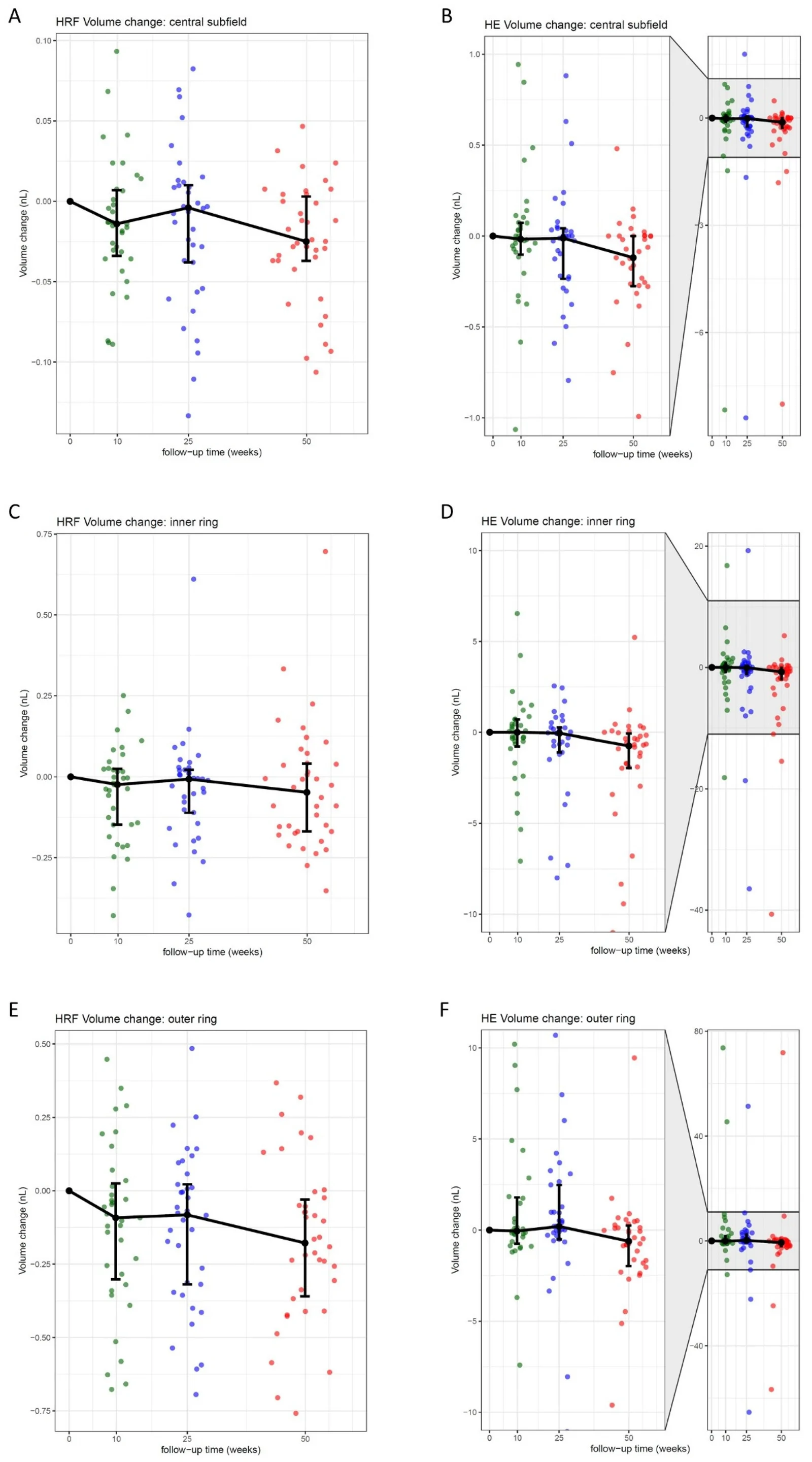
Graphs showing the change in HRF volume (**A**,**C**,**E**) and HE volume (**B**,**D**,**F**) over time in the central subfield (**A**,**B**), the inner (**C**,**D**), and the outer (**E**,**F**) ETDRS rings. Each dot represents an individual patient visit at visit 2 (green), visit 3 (blue), and visit 4 (red). To improve visualization of HE changes in panels (**B**,**D**,**F**), the corresponding portions of the graphs were magnified; the outliers remain visible on the right. The error bars show Q1 (25th percentile) and Q3 (75th percentile). Sample size was 35 at visit 2, 37 at visit 3, and 38 at visit 4.

HRF counts significantly decreased in the central, inner nasal, outer superior, inner inferior, and outer temporal subfields and in the inner and outer rings. HE counts decreased significantly in all ETDRS subfields and both rings. HRF and HE changes in the central subfield and rings are summarized in Table 2 and also illustrated in Figure 3; complete results for all subfields and time points are reported in Supplementary Tables S2–S5.

### 3.6. Correlations

HRF and HE volumes showed significant correlations with visual acuity. HRF volume in the central subfield correlated positively with visual acuity at baseline (r_s_ = 0.51, *p* = 0.00063), at visit 3 (r_s_ = 0.48, *p* = 0.0025), and at visit 4 (r_s_ = 0.4, *p* = 0.0098). HE volume in the central subfield also correlated with visual acuity at baseline (r_s_ = 0.31, *p* = 0.049), visit 3 (r_s_ = 0.49, *p* = 0.0016), and visit 4 (r_s_ = 0.5, *p* = 0.001).

Furthermore, HRF and HE volumes were significantly associated with CST. HRF volume in the central subfield correlated with CST at all visits (baseline: r_s_ = 0.66, *p* < 0.0001; visit 2: r_s_ = 0.5, *p* = 0.0015; visit 3: r_s_ = 0.55, *p* = 0.00027; visit 4: r_s_ = 0.45, *p* = 0.0033). HE volume in the central subfield showed similar correlations (baseline: r_s_ = 0.4, *p* = 0.0099; visit 3: r_s_ = 0.51, *p* = 0.0008; visit 4: r_s_ = 0.42, *p* = 0.0067).

No significant correlation was found between total HRF/HE volume and the number of faricimab injections. Full correlation data are shown in Supplementary Table S6.

### 3.7. Morphologic Responders and Non-Responders

At visit 2, 22 eyes were categorized as morphologic responders, and 19 eyes as non-responders.

Baseline median CST was 375 µm (344; 420.5) in eyes later classified as non-responders and 321 µm (283.25; 405.25) in responders. At visit 4, CST was 315 µm (288; 332) in non-responders and 279 µm (252; 326) in responders.

Median BCVA at baseline was 0.30 logMAR (0.20; 0.50) in non-responders and 0.25 logMAR (0.20; 0.38) in responders. At visit 4, BCVA was 0.20 logMAR (0.15; 0.30) in non-responders and 0.20 logMAR (0.10; 0.27) in responders.

HRF and HE volumes were available at baseline in 38 patients (18 non-responders, 20 responders) and at visit 4 in 40 patients (19 non-responders, 21 responders). For total HRF volume, baseline median volume was 1.22 nL (0.83; 1.66) in non-responders and 1.07 nL (0.78; 1.64) in responders. At visit 4, total HRF volume was 0.93 nL (0.75; 1.33) in non-responders and 0.91 nL (0.57; 1.19) in responders.

Central subfield median HRF volume was 0.06 nL (0.03; 0.10) in non-responders and 0.04 nL (0.03; 0.07) in responders at baseline, 0.03 nL (0.02; 0.05) and 0.03 nL (0.01; 0.04) at visit 4, respectively. In the inner ring, baseline HRF volume was 0.28 nL (0.20; 0.46) in non-responders and 0.28 nL (0.16; 0.39) in responders. At visit 4, volumes were 0.29 nL (0.17; 0.48) and 0.21 nL (0.11; 0.33). In the outer ring, baseline volumes were 0.79 nL (0.41; 0.90) and 0.61 nL (0.47; 0.95), and visit 4 values were 0.52 nL (0.36; 0.65) and 0.50 nL (0.29; 0.81) in non-responders and responders, respectively.

For total HE volume, medians were 6.62 nL (3.73; 14.11) in non-responders and 4.55 nL (1.48; 8.04) in responders at baseline and 3.19 nL (1.18; 9.68) in non-responders and 2.33 nL (0.79; 5.26) in responders at visit 4.

Central subfield HE volume was 0.27 nL (0.03; 0.57) in non-responders and 0.23 nL (0.04; 0.38) in responders at baseline, and 0.06 nL (0.03; 0.23) and 0.03 nL (0; 0.18) at visit 4, respectively. In the inner ring, baseline HE volume was 3.04 nL (1.32; 4.16) in non-responders and 0.88 nL (0.48; 2.50) in responders. At visit 4, volumes were 1.20 nL (0.53; 2.17) and 0.64 nL (0.15; 1.18). In the outer ETDRS ring, baseline values were 2.93 nL (0.99; 8.01) and 1.86 nL (0.40; 4.71), and visit 4 values were 1.36 nL (0.38; 4.53) and 1.14 nL (0.42; 4.01) in non-responders and responders, respectively.

## 4. Discussion

This single-center real-world study provides a longitudinal volumetric analysis of HRF and HE in eyes with DME switched from aflibercept to faricimab. HRF and HE volumes and counts decreased during one year of follow-up, with the most pronounced changes observed at the final visit.

Previous studies have demonstrated reductions in HRF and HE during anti-VEGF treatment using different imaging endpoints. Schreur et al. reported a reduction in HRF counts following bevacizumab [17], while Huang et al. observed decreasing HRF counts across retinal layers over one year of treatment with ranibizumab or aflibercept [18]. Regarding HE, Srinivas et al. demonstrated a gradual reduction in two-dimensional HE area during ranibizumab treatment [10]. Moreover, studies investigating the influence of intravitreal corticosteroid therapy have shown a decrease in HE area throughout treatment [15,19,20].

Nevertheless, it is important to consider the methodological approaches used to assess these changes in HRF and HE. Traditionally, HE area has been quantified using two-dimensional fundus photography [19]. However, this method may not fully capture the complexity of exudative lesions. Kim et al. demonstrated the feasibility and effectiveness of OCT for volumetric quantification of HEs in DME, showing moderate correlation with conventional area-based measurements while offering a more comprehensive assessment [21]. A recent analysis of Protocol T showed a substantial reduction in OCT-derived HE volume at one year during ranibizumab, bevacizumab, or aflibercept treatment [22], consistent with the reduction in HE volume in our study. Volumetric analysis may provide additional information by capturing lesion depth, thickness, and overall volume, which are not represented by two-dimensional measurements.

We did not subclassify HRF by presumed origin, as our aim was to quantify overall HRF burden and its association with anatomical and functional treatment response. However, recent work by Frizziero et al. showed that HRF may have heterogeneous origins, deriving from activated microglia, migrating retinal pigment epithelium, vascular-associated elements, or lipid exudation [4]. This diverse cellular origin underscores their complexity as biomarkers and their potential role in indicating different pathophysiological processes.

Building on the understanding of HRF as markers of inflammation and vascular instability, faricimab’s dual inhibition of both VEGF-A and Angiopoietin-2 (Ang-2) makes it a promising treatment option in DME. Preclinical studies show that Ang-2 promotes retinal vascular leakage and endothelial inflammatory responses, whereas Ang-2 neutralization reduces leakage in the diabetic retina, providing a potential mechanism for the observed HRF and HE reductions [23,24,25].

In a YOSEMITE and RHINE post hoc analysis investigating the volumetric dynamics of HRF, a greater reduction in HRF volume was reported with faricimab compared to aflibercept [26]. This trend was particularly evident by week 48, where our results mirrored the patterns of improvement of the faricimab treatment arm. However, we did not observe the early transient increase in HRF volume within the first weeks that was noted in the YOSEMITE/RHINE data. The absence of a comparable early increase in our cohort may be related to differences in prior treatment exposure, because all eyes in the present study had previously received aflibercept.

Nonetheless, the directionally similar findings at later time points in our cohort and in the YOSEMITE/RHINE analysis support further investigation of HRF as a potential imaging biomarker for disease activity and treatment response in DME.

In addition to the longitudinal reductions, the correlation analyses provide context for the potential clinical relevance of regional HRF and HE quantification. At time points with statistically significant associations, greater central HRF and HE volumes were associated with poorer visual acuity, as indicated by positive correlations with BCVA. Central HRF volume also showed moderate to strong correlations with CST, whereas the correlations of central HE volume with both BCVA and CST were generally moderate. These findings suggest that central lesion burden may reflect both anatomical and functional disease severity. Notably, the presence of HE within the central fovea is of particular concern, given its critical role in high-resolution vision. Exudates in the foveal center may obstruct the optical pathway or trigger localized inflammation and structural damage, ultimately leading to a measurable decline in visual acuity. Raman et al. demonstrated reduced retinal sensitivity at sites of HE accumulation using microperimetry measurements [27]. These findings are further supported by a recent study by Stino et al. [28], which found a correlation between HE volume and retinal sensitivity. While Raman et al. did not detect a correlation between HE area and retinal sensitivity, the results from Stino et al. suggest that volumetric measurements may provide information about HE burden that is not fully captured by area-based measurements.

In our response-stratified analysis, the early CST-based classification primarily captured differences in retinal thickness, whereas HRF and HE metrics provided additional insights into treatment response on a microstructural level over one year. Total HRF volume and regional HRF volumes decreased to a similar extent in both groups, converging to comparable levels by year one. Given that OCT hyper-reflective foci are widely regarded as in vivo biomarkers of local retinal inflammation in diabetic retinopathy and DME [4], the parallel HRF reduction in early responders and non-responders suggests that changes in HRF burden may not be fully reflected by the CST-based response classification. In contrast, total HE volume was consistently higher at baseline in eyes later classified as CST non-responders, despite all patients having been switched from aflibercept. The greater HE burden at the time of switching may reflect residual lipid deposition following prior aflibercept treatment. Nevertheless, total HE volume was approximately halved in both response groups and across macular subfields over one year, indicating a descriptive reduction in exudative burden during follow-up in both groups. Given the small subgroup sizes and the absence of confirmatory between-group testing, these findings should be considered hypothesis-generating.

Regarding regional distribution, HE showed a trend towards greater baseline burden in the superior and temporal subfields and in the outer ETDRS ring. The relative distribution of HE and HRF volume changed only marginally during follow-up, indicating that the regional pattern remained broadly stable despite the overall volumetric reductions. The larger absolute changes in the outer ring, particularly in the temporal subfield, may therefore partly reflect the greater baseline lesion burden, although the larger area of the outer ring may also contribute. Similar radial patterns have been reported previously: Protocol T demonstrated substantially greater baseline HE volume and the largest absolute reduction in the outer ring [22], while Pemp et al. described HE predominantly in perifoveal regions, particularly at the borders of clinically significant macular edema [29]. Von Schulthess et al. likewise found greater HRF burden within the 3 mm ETDRS diameter than in the central subfield and reported that HRF distribution closely followed that of intraretinal fluid [30]. However, longitudinal HE and HRF changes across the individual superior, inferior, nasal, and temporal ETDRS subfields have not been systematically compared, and our findings provide additional information on this directional distribution.

Volumetric HRF and HE quantification complements conventional OCT assessment by providing three-dimensional and region-specific measures of lesion burden. The median BCVA improvement of 0.10 logMAR and CST reduction of 48 µm at 1 year provide conventional functional and anatomical context for these imaging changes, although thresholds for clinically meaningful HRF and HE volume changes have not yet been established. At present, volumetric quantification may be most relevant as a standardized regional imaging outcome in clinical research. Prospective studies should determine whether it provides predictive information beyond conventional OCT parameters or influences treatment decisions. The limitations of this study include its retrospective design and relatively small sample size. Additionally, no adjustments were made for potential confounding factors. The absence of a control group and the previous exposure of all eyes to aflibercept prevent attribution of the observed changes specifically to faricimab or to dual VEGF-A/Ang-2 inhibition. In addition, numerous comparisons were conducted across subfields, visits, imaging outcomes, and correlation analyses without adjustment for multiple testing, increasing the risk of false-positive findings. The responder subgroup analyses were descriptive and involved small numbers of eyes. Accordingly, the statistical findings should be interpreted as exploratory and hypothesis-generating. Despite manual review and correction, residual segmentation or lesion-classification errors cannot be excluded. Furthermore, the internally developed segmentation algorithm and annotation software are not publicly available, which may limit independent replication of the imaging workflow. To further validate the utility of HRF and HE as biomarkers for treatment response in DME, larger, prospective studies are warranted.

Together, our findings show that HRF and HE volumes decreased during one year of faricimab treatment in previously treated eyes with DME. Simultaneous volumetric analysis of both biomarkers characterized regional and longitudinal changes in lesion burden and supports their further prospective evaluation as imaging biomarkers in DME.

## Figures and Tables

**Table 1:** Patient characteristics at baseline. Categorical variables are reported as counts (N) and metric variables are reported as median (Q1; Q3).

Baseline Characteristics	
Number of patients/number of eyes	44/44
Patient age (years)	64 (57; 72)
Gender (female/male)	28/16
Diabetes mellitus type (1/2)	6/38
Insulin-dependency (no/yes/missing)	13/18/13
Time since diabetes diagnosis (years)	15 (9.25; 22.25), missing: 4
Last known HbA1c (%)	7.1 (6.5; 8), missing: 4
Diabetic retinopathy (DR) stage (N)	
- mild non-proliferative DR (NPDR)	3
- moderate NPDR	16
- severe NPDR	14
- proliferative DR	6
- quiescent proliferative DR	5
BCVA (LogMAR)	0.3 (0.2; 0.43)
CST (µm)	351.5 (303; 421.75)
Number of prior intravitreal anti-VEGF injections	11 (6; 16)
Prior ocular Surgery (multiple categories per case possible)	
- Panretinal lasercoagulation	14
- macular lasercoagulation	2
- cataract surgery	21
- vitrectomy	1

**Table 2:** Difference from baseline of HRF and HE volume (nL) and counts (*n*). Median difference to baseline, as well as the first (Q1) and third (Q3) quartiles, are reported in nL (volume) and absolute counts (count). Statistically significant results (*p* < 0.05) are highlighted in bold font. The sample size was 35 at visit 2, 37 at visit 3, and 38 at visit 4.

Variable	Area	Visit	Median Difference to Baseline	Q1	Q3	*p* Value
HRF volume (nL)	Central	2	−0.014	−0.034	0.007	**0.038**
		3	−0.004	−0.038	0.010	0.097
		4	−0.025	−0.037	0.003	**0.00028**
	Inner ring	2	−0.024	−0.148	0.025	0.062
		3	−0.007	−0.111	0.022	0.11
		4	−0.048	−0.169	0.041	0.079
	Outer ring	2	−0.092	−0.302	0.025	**0.018**
		3	−0.082	−0.319	0.022	**0.01**
		4	−0.178	−0.360	−0.030	**0.00041**
HRF count (*n*)	Central	2	−2.0	−6.00	0.50	**0.016**
		3	−2.0	−8.00	0.00	**0.022**
		4	−4.0	−6.75	0.00	**0.00031**
	Inner ring	2	−3.0	−29.00	2.00	**0.015**
		3	−6.0	−22.00	8.00	0.06
		4	−7.5	−32.00	7.75	**0.022**
	Outer ring	2	−11.0	−62.00	10.00	0.054
		3	−15.0	−51.00	9.00	**0.021**
		4	−24.5	−57.75	1.00	**0.0054**
HE volume (nL)	Central	2	−0.017	−0.102	0.073	0.54
		3	−0.012	−0.235	0.042	0.26
		4	−0.119	−0.276	0.000	**0.00025**
	Inner ring	2	0.000	−0.762	0.715	0.73
		3	−0.048	−1.093	0.270	0.25
		4	−0.740	−1.951	−0.063	**<0.0001**
	Outer ring	2	−0.055	−0.747	1.793	0.54
		3	0.201	−0.510	2.481	0.31
		4	−0.612	−1.978	0.245	**0.014**
HE count (*n*)	Central	2	−1.0	−4.50	1.00	**0.039**
		3	−1.0	−5.00	0.00	0.052
		4	−1.5	−5.00	0.00	**0.0026**
	Inner ring	2	0.0	−9.50	6.00	0.36
		3	0.0	−9.00	5.00	0.52
		4	−9.0	−22.75	0.00	**0.0012**
	Outer ring	2	1.0	−13.00	16.00	0.88
		3	−1.0	−12.00	8.00	0.36
		4	−16.0	−38.50	−0.25	**0.00053**

## Data Availability

The datasets analyzed during the current study are not publicly available due to the inclusion of sensitive patient data and applicable data protection regulations but are available from the corresponding author on reasonable request, subject to approval by the relevant institutional review board and in accordance with applicable data protection and ethical requirements.

## Supplementary Materials

The following supporting information can be downloaded at: https://www.mdpi.com/article/10.3390/jcm15166319/s1. Table S1: Baseline measures of HRF and HE volume and count. For the volumetric variables, the median as well as the first (Q1) and third (Q3) quartiles are reported in nL. For the ‘number’ variables, the median as well as the first (Q1) and third (Q3) quartiles are reported as absolute counts. The sample size was 41. Table S2: Difference from baseline of HRF volumes. Median difference to baseline, as well as the first (Q1) and third (Q3) quartiles, are reported in nL. Statistically significant results (*p* < 0.05, Wilcoxon signed-rank test) are highlighted in bold font. The sample size was 35 at visit 2, 37 at visit 3 and 38 at visit4. Table S3: Difference from baseline of HE volume. Median difference to baseline, as well as the first (Q1) and third (Q3) quartiles, are reported in nL. Statistically significant results (*p* < 0.05, Wilcoxon signed-rank test) are highlighted in bold font. The sample size was 35 at visit 2, 37 at visit 3 and 38 at visit4. Table S4: Difference from baseline of HRF counts. Median difference to baseline, as well as the first (Q1) and third (Q3) quartiles, are reported in absolute counts. Statistically significant results (*p* < 0.05, Wilcoxon signed-rank test) are highlighted in bold font. The sample size was 35 at visit 2, 37 at visit 3 and 38 at visit4. Table S5: Difference from baseline of HE counts. Median difference to baseline, as well as the first (Q1) and third (Q3) quartiles, are reported in absolute counts. Statistically significant results (*p* < 0.05, Wilcoxon signed-rank test) are highlighted in bold font. The sample size was 35 at visit 2, 37 at visit 3 and 38 at visit4. Table S6: Correlation analyses. Spearman correlation coefficients (rs) are reported along with *p*-values for statistical significance. Correlations with a Spearman coefficient of >0.3 and >0.6 are considered moderate and strong, respectively. Statistically significant results (*p* < 0.05, Wilcoxon signed-rank test) are highlighted in bold font. Sample size for the correlation with BCVA was (Baseline/visit 2/visit 3/visit 4) 41/37/38/40 and for the correlation with CST, 41/37/39/40. Sample size for the correlation between HRF (or HE) volume and number of injections was *n* = 40.
