# Supplementary material for: Dynamics of Hyper-Reflective Foci and Hard Exudates in Diabetic Macular Edema Treated with Faricimab
Source: J Clin Med. 2026 Aug 15;15(16):6319. doi: 10.3390/jcm15166319 (PMC13513130; doi:10.3390/jcm15166319)
Supplement: Supplementary file 1 [file jcm-15-06319-s001.zip › jcm-4466018-supplementary.pdf]

Dynamics of hyperreflective foci and hard exudates in diabetic macular edema treated with faricimab

Paul Widmann-Sedlnitzky, Kim Lien Huber, Irene Steiner, Markus Gumpinger, Heiko Stino, Tilman Schmoll, Ursula Schmidt-Erfurth, Stefan Sacu, Andreas Pollreisz

Supplement:

Variable	area	median	Q1	Q3
HRF volume	central	0.05	0.03	0.08
	inner ring	0.28	0.19	0.44
	outer ring	0.61	0.40	0.92
	inner nasal	0.06	0.01	0.09
	outer nasal	0.11	0.07	0.20
	inner superior	0.07	0.03	0.12
	outer superior	0.14	0.07	0.26
	inner inferior	0.05	0.03	0.10
	outer inferior	0.12	0.07	0.21
	inner temporal	0.09	0.04	0.16
	outer temporal	0.19	0.11	0.35
HRF number	central	10.00	5.00	13.00
	inner ring	54.00	39.00	77.00
	outer ring	126.00	84.00	166.00
	inner nasal	11.00	4.00	18.00
	outer nasal	23.00	16.00	38.00
	inner superior	13.00	7.00	24.00
	outer superior	25.00	15.00	46.00
	inner inferior	12.00	6.00	18.00

Variable	area	median	Q1	Q3
	outer inferior	27.00	13.00	42.00
	inner temporal	18.00	9.00	27.00
	outer temporal	40.00	26.00	55.00
HE volume	central	0.21	0.03	0.39
	inner ring	1.54	0.75	3.64
	outer ring	2.91	0.66	7.83
	inner nasal	0.20	0.00	0.63
	outer nasal	0.12	0.02	0.52
	inner superior	0.25	0.04	0.55
	outer superior	0.20	0.04	1.75
	inner inferior	0.22	0.03	0.78
	outer inferior	0.35	0.03	0.94
	inner temporal	0.38	0.17	1.48
	outer temporal	0.74	0.12	1.85
HE number	central	4.00	1.00	9.00
	inner ring	27.00	10.00	48.00
	outer ring	49.00	14.00	89.00
	inner nasal	4.00	0.00	8.00
	outer nasal	4.00	1.00	11.00
	inner superior	4.00	1.00	15.00
	outer superior	7.00	2.00	28.00
	inner inferior	4.00	1.00	11.00
	outer inferior	9.00	2.00	17.00
	inner temporal	8.00	3.00	15.00
	outer temporal	14.00	3.00	27.00

Supplementary Table S1: Baseline measures of HRF and HE volume and count. For the volumetric variables, the median as well as the first (Q1) and third (Q3) quartiles are reported in nL. For the 'number' variables, the median as well as the first (Q1) and third (Q3) quartiles are reported as absolute counts. The sample size was 41.

Variable	area	Visit	median	Q1	Q3	p-value
Difference from baseline, HRF volume	central	2	-0.014	-0.034	0.007	**0.038**
		3	-0.004	-0.038	0.010	0.097
		4	-0.025	-0.037	0.003	**0.00028**
	inner ring	2	-0.024	-0.148	0.025	0.062
		3	-0.007	-0.111	0.022	0.11
		4	-0.048	-0.169	0.041	0.079
	outer ring	2	-0.092	-0.302	0.025	**0.018**
		3	-0.082	-0.319	0.022	**0.01**
		4	-0.178	-0.360	-0.030	**0.00041**
	outer nasal	2	-0.007	-0.078	0.016	0.08
		3	-0.022	-0.064	0.029	0.12
		4	-0.034	-0.092	-0.001	**0.0097**
	inner nasal	2	-0.007	-0.046	0.024	0.22
		3	-0.004	-0.040	0.012	0.22
		4	-0.001	-0.052	0.016	0.14
	outer superior	2	-0.021	-0.048	0.027	0.2
		3	-0.015	-0.079	0.015	0.054
		4	-0.053	-0.096	-0.008	**0.0037**
	inner superior	2	-0.012	-0.034	0.017	0.17
		3	0.003	-0.036	0.014	0.43
		4	-0.005	-0.033	0.024	0.33
	outer inferior	2	-0.028	-0.068	0.040	0.42
		3	0.000	-0.056	0.033	0.7
		4	-0.020	-0.069	0.018	0.073
	inner inferior	2	-0.005	-0.024	0.015	0.27
		3	-0.002	-0.027	0.014	0.51
		4	-0.010	-0.035	0.009	0.1
	outer temporal	2	-0.034	-0.116	0.000	**0.0025**
		3	-0.018	-0.155	0.029	**0.017**
		4	-0.028	-0.128	0.016	**0.0081**
	inner temporal	2	-0.021	-0.062	0.008	**0.045**
		3	-0.027	-0.061	0.014	0.03
		4	-0.014	-0.065	0.037	0.2

Supplementary Table S2: Difference from baseline of HRF volumes. Median difference to baseline, as well as the first (Q1) and third (Q3) quartiles, are reported in nL. Statistically significant results ($p < 0.05$, Wilcoxon signed-rank test) are highlighted in bold font. The sample size was 35 at visit 2, 37 at visit 3 and 38 at visit4.

Variable	area	Visit	median	Q1	Q3	p-value
Difference from baseline, HE volume	central	2	-0.017	-0.102	0.073	0.54
		3	-0.012	-0.235	0.042	0.26
		4	-0.119	-0.276	0.000	**0.00025**
	inner ring	2	0.000	-0.762	0.715	0.73
		3	-0.048	-1.093	0.270	0.25
		4	-0.740	-1.951	-0.063	**<0.0001**
	outer ring	2	-0.055	-0.747	1.793	0.54
		3	0.201	-0.510	2.481	0.31
		4	-0.612	-1.978	0.245	**0.014**
	outer nasal	2	-0.015	-0.059	0.297	0.82
		3	-0.014	-0.195	0.039	0.51
		4	-0.031	-0.287	0.028	0.091
	inner nasal	2	0.000	-0.104	0.141	0.78
		3	-0.014	-0.231	0.024	0.16
		4	-0.040	-0.225	0.040	0.1
	outer superior	2	0.079	-0.107	0.339	0.24
		3	0.024	-0.082	0.394	0.55
		4	-0.042	-0.506	0.033	0.054
	inner superior	2	0.016	-0.129	0.232	0.51
		3	0.000	-0.178	0.158	0.81
		4	-0.042	-0.232	0.002	**0.035**
	outer inferior	2	0.001	-0.128	0.123	0.76
		3	-0.012	-0.224	0.056	0.63
		4	-0.111	-0.426	0.000	**0.002**
	inner inferior	2	-0.033	-0.387	0.137	0.42
		3	-0.033	-0.355	0.054	0.072
		4	-0.136	-0.727	0.000	**<0.0001**
	outer temporal	2	0.011	-0.287	0.735	0.28
		3	0.000	-0.620	0.209	0.65
		4	-0.116	-1.050	0.066	**0.041**
	inner temporal	2	-0.018	-0.397	0.121	0.18
		3	-0.034	-0.477	0.105	0.1
		4	-0.239	-0.649	0.014	**0.0017**

Supplementary Table S3: Difference from baseline of HE volume. Median difference to baseline, as well as the first (Q1) and third (Q3) quartiles, are reported in nL. Statistically significant results ($p < 0.05$, Wilcoxon signed-rank test) are highlighted in bold font. The sample size was 35 at visit 2, 37 at visit 3 and 38 at visit4.

Variable	area	Visit	median	Q1	Q3	p-value
Difference from baseline, HRF number	central	2	-2.0	-6.00	0.50	**0.016**
		3	-2.0	-8.00	0.00	**0.022**
		4	-4.0	-6.75	0.00	**0.00031**
	inner ring	2	-3.0	-29.00	2.00	**0.015**
		3	-6.0	-22.00	8.00	0.06
		4	-7.5	-32.00	7.75	**0.022**
	outer ring	2	-11.0	-62.00	10.00	0.054
		3	-15.0	-51.00	9.00	**0.021**
		4	-24.5	-57.75	1.00	**0.0054**
	outer nasal	2	-4.0	-16.00	3.50	**0.036**
		3	-2.0	-12.00	7.00	0.35
		4	-3.5	-15.00	7.00	0.11
	inner nasal	2	-2.0	-6.00	3.00	0.12
		3	-2.0	-7.00	1.00	**0.034**
		4	-3.0	-7.00	2.00	**0.03**
	outer superior	2	-2.0	-14.00	6.00	0.34
		3	-3.0	-15.00	4.00	**0.028**
		4	-9.0	-15.75	-1.00	**0.005**
	inner superior	2	-2.0	-8.00	3.00	**0.043**
		3	0.0	-8.00	3.00	0.2
		4	-3.0	-8.75	3.75	0.083
	outer inferior	2	-2.0	-12.00	4.50	0.25
		3	-1.0	-9.00	6.00	0.69
		4	-4.0	-11.00	5.00	0.14
	inner inferior	2	-1.0	-6.00	2.00	**0.049**
		3	-1.0	-6.00	4.00	0.3
		4	-2.5	-8.25	2.75	**0.043**
	outer temporal	2	-4.0	-24.00	1.50	**0.021**
		3	-4.0	-26.00	4.00	**0.023**
		4	-6.0	-18.75	0.00	**0.0091**
	inner temporal	2	-6.0	-10.50	1.50	**0.031**
		3	-4.0	-9.00	7.00	0.13
		4	-2.0	-9.75	6.75	0.18

Supplementary Table S4: Difference from baseline of HRF counts. Median difference to baseline, as well as the first (Q1) and third (Q3) quartiles, are reported in absolute counts. Statistically significant results ($p < 0.05$, Wilcoxon signed-rank test) are highlighted in bold font. The sample size was 35 at visit 2, 37 at visit 3 and 38 at visit4.

Variable	area	Visit	median	Q1	Q3	p-value
Difference from baseline, HE number	central	2	-1.0	-4.50	1.00	**0.039**
		3	-1.0	-5.00	0.00	0.052
		4	-1.5	-5.00	0.00	**0.0026**
	inner ring	2	0.0	-9.50	6.00	0.36
		3	0.0	-9.00	5.00	0.52
		4	-9.0	-22.75	0.00	**0.0012**
	outer ring	2	1.0	-13.00	16.00	0.88
		3	-1.0	-12.00	8.00	0.36
		4	-16.0	-38.50	-0.25	**0.00053**
	outer nasal	2	-1.0	-1.50	3.00	0.72
		3	0.0	-3.00	0.00	0.21
		4	-2.0	-8.50	0.00	**0.0017**
	inner nasal	2	0.0	-3.00	1.00	0.073
		3	0.0	-4.00	1.00	0.22
		4	-1.5	-5.75	1.00	**0.013**
	outer superior	2	0.0	-4.50	4.50	0.92
		3	0.0	-2.00	5.00	0.83
		4	-2.0	-7.00	0.75	**0.024**
	inner superior	2	0.0	-2.00	2.50	0.95
		3	0.0	-5.00	1.00	0.57
		4	-1.5	-6.00	1.00	**0.041**
	outer inferior	2	0.0	-4.50	4.50	0.95
		3	-1.0	-4.00	2.00	0.16
		4	-2.5	-7.50	0.00	**0.0011**
	inner inferior	2	0.0	-3.00	1.00	0.3
		3	0.0	-3.00	1.00	0.37
		4	-3.0	-7.00	0.00	**0.00023**
	outer temporal	2	-1.0	-6.00	1.50	0.29
		3	0.0	-8.00	2.00	0.084
		4	-5.0	-13.75	1.00	**0.0047**
	inner temporal	2	0.0	-5.00	2.00	0.34
		3	-1.0	-5.00	2.00	0.1
		4	-2.5	-7.00	1.00	**0.019**

Supplementary Table S5: Difference from baseline of HE counts. Median difference to baseline, as well as the first (Q1) and third (Q3) quartiles, are reported in absolute counts. Statistically significant results (p < 0.05, Wilcoxon signed-rank test) are highlighted in bold font. The sample size was 35 at visit 2, 37 at visit 3 and 38 at visit4.

Correlation variables		Baseline	8	24 ± 4	50 ± 10
HRF volume central - BCVA	rs	0.51	0.32	0.48	0.4
	p-value	**0.00063**	0.051	**0.0025**	**0.0098**
HRF count central - BCVA	rs	0.49	0.34	0.45	0.34
	p-value	**0.001**	**0.037**	**0.0045**	**0.029**
HE volume central - BCVA	rs	0.31	0.3	0.49	0.5
	p-value	**0.049**	0.07	**0.0016**	**0.001**
HE count central - BCVA	rs	0.42	0.18	0.47	0.56
	p-value	**0.0063**	0.28	**0.0027**	**0.00018**
HRF volume central - CST	rs	0.66	0.5	0.55	0.45
	p-value	**< 0.0001**	**0.0015**	**0.00027**	**0.0033**
HRF count central - CST	rs	0.65	0.54	0.57	0.36
	p-value	**< 0.0001**	**0.00063**	**0.00013**	**0.021**
HE volume central - CST	rs	0.4	0.16	0.51	0.42
	p-value	**0.0099**	0.34	**0.0008**	**0.0067**
HE count central - CST	rs	0.49	0.39	0.55	0.56
	p-value	**0.0013**	**0.016**	**0.00025**	**0.00018**
HRF volume inside 6mm ring - number of injections	rs				0.253
	p-value				0.11
HE volume inside 6mm ring - number of injections	rs				0.157
	p-value				0.33

Supplementary Table S6: Correlation analyses. Spearman correlation coefficients (rs) are reported along with p-values for statistical significance. Correlations with a Spearman coefficient of >0.3 and >0.6 are considered moderate and strong, respectively. Statistically significant results (p < 0.05, Wilcoxon signed-rank test) are highlighted in bold font. Sample size for the correlation with BCVA was (Baseline/ visit 2/ visit 3/ visit 4) 41/ 37/ 38/ 40 and for the correlation with CST, 41/ 37/ 39/ 40. Sample size for the correlation between HRF (or HE) volume and number of injections was n = 40.
